# Baseline Body Composition and Physical Activity Level Recommended for Optimal Bone Mineral Density in Young Women

**DOI:** 10.1089/whr.2021.0137

**Published:** 2022-03-21

**Authors:** Sangun Lee, Chikako Fujita, Atsuko Satoh

**Affiliations:** ^1^ Department of Physical Therapy, Faculty of Health Sciences, Aomori University of Health and Welfare, Aomori, Japan.; ^2^ Aomori University of Health and Welfare Graduate School of Health Sciences, Aomori, Japan.; ^3^ Department of Nursing, Junior College, Hirosaki University of Health and Welfare, Hirosaki, Japan.

**Keywords:** bone mineral density, peak bone mass, body composition, physical activity, baseline values

## Abstract

**Aim:**

This study examined the influence of body composition and physical activity level (PAL) on bone mineral density (BMD) to determine the baseline values necessary for maintaining healthy trabecular bone and improving bone health in young women.

**Materials and Methods:**

The subjects, consisting of healthy young women, were assigned to either a BMD-low (BMD-L) or BMD-high (BMD-H) group using the osteosonic index. PAL was measured for 1 week and rated on a scale from PAL-1 to 8 according to intensity levels (metabolic equivalents [METs]). The Wilcoxon rank-sum test was performed for intergroup comparisons.

**Results:**

The BMD-H group had significantly higher fat-free muscle mass, skeletal muscle index, and basic metabolic rate than the BMD-L group (*p* < 0.001, each). Regarding the timing of physical activity in daily life according to intensity, PAL-6 (*p* < 0.01), PAL-7 (*p* < 0.001), and PAL-8 (*p* < 0.01) for the BMD-H group were significantly longer than those for the BMD-L group.

**Discussion and Conclusion:**

For young women in their early 20s, BMD may be associated with baseline physical fitness and strength, as determined by body composition, but it is not influenced by the duration of physical activity. A PAL ≥6.0 METs may improve or maintain the effect on BMD.

## Introduction

Bedridden people and astronauts who stay in space for an extended period are known to experience trabecular bone loss and disuse atrophy.^1–3^ Existing findings suggest that internal and external mechanical stress (MS) are important for bones, and high-impact loads are more effective.^4–6^ However, bones require MS above a certain intensity level and sufficient rest, which is supported by Wolff's law and the mechanostat theory.^7,8^ Specifically, bones have a certain MS threshold; MS exceeding the threshold and adequate rest are important for maintaining healthy trabecular bones and improving bone health.

Physical activity (PA) is affected by weight, muscle mass, and movement in the form of walking or running, and it is influenced by body composition, including the length of the lower limbs and muscular strength and mass.^9–12^ Lifestyle, muscle power, and exercise history may also affect PA.^13–15^ Groothausen et al.^16^ classified four stages of load during sports activities based on the mechanical components of PA, suggesting the involvement of PA and bone mineral density (BMD). In other words, the characteristics of body composition and PA possibly cause a change in the characteristics of the MS affecting the bones.

Body composition has a major effect on bones,^17–22^ and its composition depends on the characteristics and frequency of the exercise performed.^23–28^ Load placed on the body during PA is involved in energy metabolism within the organism, and mainly, the load from aerobic exercises decreases lipid-related body composition.^23,24^ In contrast, high-load resistance training affects body composition such as muscle mass and lean mass associated with muscle hypertrophy more than lipid-related factors do.^25–27^ Although many previous studies pertaining to bone health and loading have reported the benefits of heavy loading on bones, only a few studies have been conducted on the improvement of bone health from low-load aerobic exercise.^4–8,29^

Furthermore, excessive loading has been reported to be detrimental to bones, but no consensus has been reached regarding the daily load that is necessary for bones. Daily PA is considered an important factor for bone health. Studies on bones and daily PA, including exercise, are needed. However, although existing studies on bones and loading have mainly focused on exercise, the influence of daily PA intensity, excluding exercise, has not been clarified.

Bones reach peak bone mass (PBM) around 20 years of age but begin to decrease in mass and density due to factors such as aging, exercise habits, and nutritional status.^30–33^ Determining the baseline body composition and physical activity level (PAL) of young women who have achieved PBM could help to develop lifestyle-guiding tools that lead to PBM and optimize BMD.

Therefore, this study aimed to examine how body composition and PA strengthen bones and obtain the baseline values sought in preventative medicine to maintain healthy trabecular bones and improve bone health in young women.

## Materials and Methods

Research Ethics Approval No: 20003.

### Subjects

The subjects were recruited through posters at educational and medical institutions in Aomori Prefecture, and we measured while visiting those institutions interested in participating in the study. The sample consisted of 424 healthy young women (18–23 years old) who had no major bone diseases and no history of fracture or hospitalization for an orthopedic surgery within the past year as of the day when measurements were obtained. Furthermore, pregnant subjects and those with secondary amenorrhea were excluded, considering the influence of age and sex hormones.

Moreover, eligible subjects had not been taking nonsteroidal anti-inflammatory drugs or any supplements for bones within the past year as of the day when measurements were obtained. The methods and objectives of the study were explained to the subjects in detail, and all subjects provided written informed consent. The subjects were classified into BMD-Low (BMD-L) and BMD-High (BMD-H) groups using the Osteo-Sono Assessment Index (OSI). This study was approved by the Ethical Committee of Aomori University of Health and Welfare (Research Ethics Approval No. 20003).

### Questionnaire

The subjects' basic attributes were recorded using self-completed questionnaires and were collected to assess study eligibility. The questionnaire items included age, habits (alcohol intake, smoking, and exercise), medical history, medications, and menarcheal age. Lifestyle habits were defined by the Japanese Ministry of Health, Labour and Welfare.^34^ Drinking habit was defined as drinking 3 or more days a week (one or more cups of sake equivalent per drinking day), and smoking habit was defined as current habitual smoking. Habitual exercisers were defined as those who engaged in aerobic exercise for 30 minutes or more at a time, at least twice a week, for at least 1 year.

### Body composition

Anthropometric parameters included body weight (BW), body mass index (BMI), lean body mass (LBM), body fat mass, body fat percentage (%FAT), muscle mass, skeletal muscle mass index (SMI), muscle mass of the limbs and trunk, and basal metabolic rate (BMR); these measured were obtained using the body composition analyzer InBody 470 (InBody Co., Seoul, South Korea) using the simultaneous multifrequency impedance measurement method. To increase measurement accuracy, alcohol consumption and strenuous exercises on the day before measurement and any form of drinking or eating 2 hours before measurement were prohibited.

At the eight sites of contact between the measuring device and the body, the skin was wiped with rubbing alcohol to remove sweat and oil. The bioelectrical impedance analysis method is a rapid, radiation-free, noninvasive, and highly accurate method of measuring body composition, frequently used in epidemiological studies and clinical diagnosis of sarcopenia.^35^ For muscle mass, a high correlation of *r* = 0.99 between InBody 470 and InBody 770 and a high correlation of *r* = 0.97 between InBody 770 and dual-energy X-ray absorptiometry were reported.^36^

### Bone mineral density

Using the AOS-100SA (Hitachi Co., Tokyo, Japan), a quantitative ultrasound (QUS) bone densitometry system, we measured the calcaneal BMD of the dominant foot. Before measurement, the skin of the foot was wiped with ethanol and dried completely. Upon confirming that the measurement site of the heel was in the correct position, the transducer case was adjusted. Echo jelly was applied to the site where the heel and membrane made contact. Then, the heel and membrane were brought into close contact with each other using appropriate pressure. Furthermore, the influence of the measurement site and temperature was adjusted to improve the accuracy of the QUS.

The parameters used in the QUS were standardized using the QUS Standardization of the Japan Osteoporosis Society in 2010. Sound of speed (SOS), OSI, broadband ultrasound attenuation (BUA), and T-score (standard deviation, %) were measured. OSI was calculated using the following formula: transmission index × SOS.^2^ The AOS-100SA is a rapid, radiation-free, noninvasive, and highly accurate method of measuring body composition, frequently used in epidemiological studies and clinical diagnosis of bone density.^37^ There is a high correlation of *r* = 0.82 between AOS-100SA and DAX for bone SOS.^38^

### PA level

The Active style Pro HJA-750C and activity meter data collection software installer Ver 2.0 were used to measure PAL (Omron Healthcare Co., Kyoto, Japan). PAL was measured over 1 week. Activity meters were worn at the level of the iliac crest during the measurement period, excluding the time when the subjects were bathing and swimming. Considering that the subjects' PAL changed more rapidly than usual, the measurement period was determined, excluding long holidays and travel periods.

PAL values recorded every 10 seconds were converted to 1-minute blocks, and the daily mean was derived. The Active Pro HJA-750C activity monitor is equipped with a 3-axis accelerometer and can record activity level. The intensity levels (in metabolic equivalents: METs) were rated on a scale from PAL-1 to PAL-8, and the execution time per intensity level was calculated. The intensity levels of PAL were classified as PAL-1 (1.0–1.9 METs), PAL-2 (2.0–2.9 METs), PAL-3 (3.0–3.9 METs), PAL-4 (4.0–4.9 METs), PAL-5 (5.0–5.9 METs), PAL-6 (6.0–6.9 METs), PAL-7 (7.0–7.9 METs), and PAL-8 (>8.0 METs). Furthermore, PAL >3.0 METs was classified as exercise. The PAL is proportional to the BMD-L group and the BMD-H group in which a significant difference was observed.

### Statistical analysis

To generate baseline values of body composition and PA data associated with a healthy BMD, we excluded subjects with a young adult mean (YAM) for BMD of <80%. The mean and standard deviation were calculated using SPSS Statistics 27 (IBM Corp., Armonk, NY), and χ^2^ test were used for lifestyle habits. The Wilcoxon rank-sum test was conducted for intergroup comparison, and the statistical significance level was set at *p*-value of <0.05.

## Results

### Subjects

Among the 424 subjects, the mean OSI was 2.810 ± 0.338, which was 4.2% higher than the YAM of 2.698 ± 0.298. No subjects were <70% (criteria value for osteoporosis) of the YAM. However, two subjects (0.5%) with a YAM of <80% (OSI <2.158; standard deviation: −1.8) were excluded from analysis. The 422 individuals with an OSI of >80% (normal range) of YAM were classified into two groups: the BMD-L group, with a YAM for BMD of <100% (*n* = 168, 39.8%), and the BMD-H group, with a YAM for BMD of ≥100% (*n* = 254, 60.2%). There were no significant differences between the two groups regarding age, age at menarche, and the basic attributes of their menstrual cycles. There was a significant difference in exercise habits (χ^2^ = 14.880, df = 1, *p* < 0.001) (Table 1).

**Table 1.** Characteristics of the Subjects

	Total (***n*** = 422)	BMD-L group (***n*** = 168)	BMD-H group (***n*** = 254)	***p***		
Age, years	19.7 ± 1.26	19.8 ± 1.33	19.6 ± 1.22	0.217		
First menstruation, years	12.1 ± 1.47	12.1 ± 1.40	12.2 ± 1.51	0.731		
Menstrual period, days	29.8 ± 4.58	29.5 ± 4.14	30.1 ± 4.85	0.212		

	**Total**	**BMD-L group**	**BMD-H group**	**χ^2^**	**df**	***p***
Habit						
Drinking						
Yes	26 (6.2)	11 (2.6)	15 (3.6)	0.072	1	0.788
No	396 (93.8)	157 (37.2)	239 (56.6)			
Smoking						
Yes	6 (1.4)	2 (0.5)	4 (1.0)	0.107	1	1.000
No	416 (98.6)	166 (39.3)	250 (59.2)			
Exercise						
Yes	102 (24.2)	24 (5.7)	78 (18.5)	14.880	1	0.001
No	320 (75.8)	144 (34.1)	176 (41.7)			

Mean ± standard deviation, *n* (%), *p*-value: BMD-L group versus BMD-H group.

BMD-H, bone mineral density-high; BMD-L, bone mineral density-low.

### Baseline values of bone mineral density and body composition

Baseline values of body composition and muscle mass by site in young women with healthy BMD are shown in Table 2.

**Table 2.** Baseline Values of Bone Mineral Density and Body Composition

	Total (***n*** = 422)	BMD-L group (***n*** = 168)	BMD-H group (***n*** = 254)	***p***
Bone mineral density				
Sound of speed, m/s	1563.3 ± 22.07	1546.1 ± 12.9	1574.7 ± 19.3	0.001
BUA, dB/MHz	74.5 ± 13.63	65.2 ± 10.30	80.6 ± 12.04	0.001
OSI, dB/MHz	2.810 ± 0.338	2.513 ± 0.120	3.006 ± 0.289	0.001
Percent T-score, %	104.1 ± 12.55	93.0 ± 4.36	111.4 ± 10.72	0.001
T-score	0.37 ± 1.136	−0.63 ± 0.393	1.03 ± 0.972	0.001
Body composition				
Height, cm	158.6 ± 5.32	158.8 ± 5.83	158.5 ± 4.96	0.653
Body weight, kg	53.3 ± 6.92	52.1 ± 6.57	54.2 ± 7.01	0.002
Body mass index, kg/m^2^	21.2 ± 2.43	20.7 ± 2.27	21.6 ± 2.46	0.001
Fat mass, kg	14.8 ± 4.46	14.3 ± 4.13	15.2 ± 4.64	0.055
Percent fat mass, %	27.5 ± 5.50	27.4 ± 5.14	27.7 ± 6.04	0.634
Lean body mass, kg	38.5 ± 3.82	37.7 ± 3.77	39.0 ± 3.76	0.001
Skeletal muscle index, kg/m^2^	6.09 ± 0.53	5.96 ± 0.50	6.18 ± 0.53	0.001
Basal metabolic rate, Kcal	1201.3 ± 82.8	1185.0 ± 81.5	1212.7 ± 81.9	0.001
Segmental muscle mass, kg				
Upper limb				
Right	1.74 ± 0.27	1.70 ± 0.25	1.78 ± 0.26	0.001
Left	1.70 ± 0.27	1.65 ± 0.26	1.73 ± 0.26	0.001
Trunk	16.6 ± 1.69	16.4 ± 1.66	16.9 ± 1.66	0.001
Lower limb				
Right	5.97 ± 0.75	5.89 ± 0.75	6.04 ± 0.74	0.035
Left	5.96 ± 0.74	5.88 ± 0.74	6.03 ± 0.74	0.037

Mean ± standard deviation, BUA, broadband ultrasound attenuation, OSI, Osteo-Sono Assessment Index, *p*-value: BMD-L group versus BMD-H group.

The BMD-H group had significantly higher values of SOS (1.8%), BUA (23.6%), OSI (19.6%), and T-score (63.5%) than the BMD-L group (*p* < 0.001, each).

The BMD-H group had significantly higher BW by 4.0% (*p* < 0.01), BMI by 4.3% (*p* < 0.001), LBM by 3.4% (*p* < 0.001), SMI by 3.7% (*p* < 0.001), and BMR by 2.3% (*p* < 0.001) than the BMD-L group. In addition, muscle mass by region was significantly higher in the BMD-H group than the BMD-L group, by 4.7% (*p* < 0.001) in the right upper limb, 4.8% (*p* < 0.001) in the left upper limb, 2.5% (*p* < 0.05) in the right lower limb, 2.6% (*p* < 0.05) in the left lower limb, and 3.0% (*p* < 0.001) in the trunk.

### Baseline values for calorie expenditure and PA level

The baseline values for calorie expenditure and intensity of PA in young women with healthy BMD are shown in Table 3. There were no significant differences between the two groups in terms of the number of steps or time spent walking daily over 1 week. However, the amount of exercise-induced calorie expenditure associated with walking was significantly higher in the BMD-H group than the BMD-L group at 11.2% (*p* < 0.05) and 11.0% (*p* < 0.05), respectively, whereas the total physical activity (8.7%, *p* < 0.05) and total calorie expenditure (3.3%, *p* < 0.01) was also significantly higher in the BMD-H group.

**Table 3.** Baseline Values for Calorie Expenditure and Physical Activity level

	Total (***n*** = 422)	BMD-L group (***n*** = 168)	BMD-H group (***n*** = 254)	***p***
Number of steps, steps	7371.7 ± 2904.2	7157.9 ± 2750.2	7540.1 ± 2992.7	0.186
Time of walking, minutes	98.5 ± 36.65	96.6 ± 36.27	100.1 ± 36.76	0.324
Physical activity, Ex				
Exercise^a^	3.44 ± 1.786	3.22 ± 1.468	3.58 ± 1.958	0.030
Activity^b^	2.31 ± 0.990	2.24 ± 0.892	2.36 ± 1.048	0.235
Total	5.74 ± 2.333	5.46 ± 1.889	5.94 ± 2.564	0.027
Calorie, Kcal				
Exercise^a^	218.5 ± 97.80	205.0 ± 80.78	227.5 ± 107.08	0.015
Activity^b^	412.8 ± 96.80	401.6 ± 90.30	420.2 ± 100.37	0.053
Total	631.4 ± 160.62	606.6 ± 136.53	647.7 ± 173.07	0.010

Mean ± standard deviation.

^a^ Exercise (>3.0 METs).

^b^ Activity (1.0–2.9 METs), *p*-value: BMD-L group versus BMD-H group.

### Strength level of PA

**FIG. 1.**
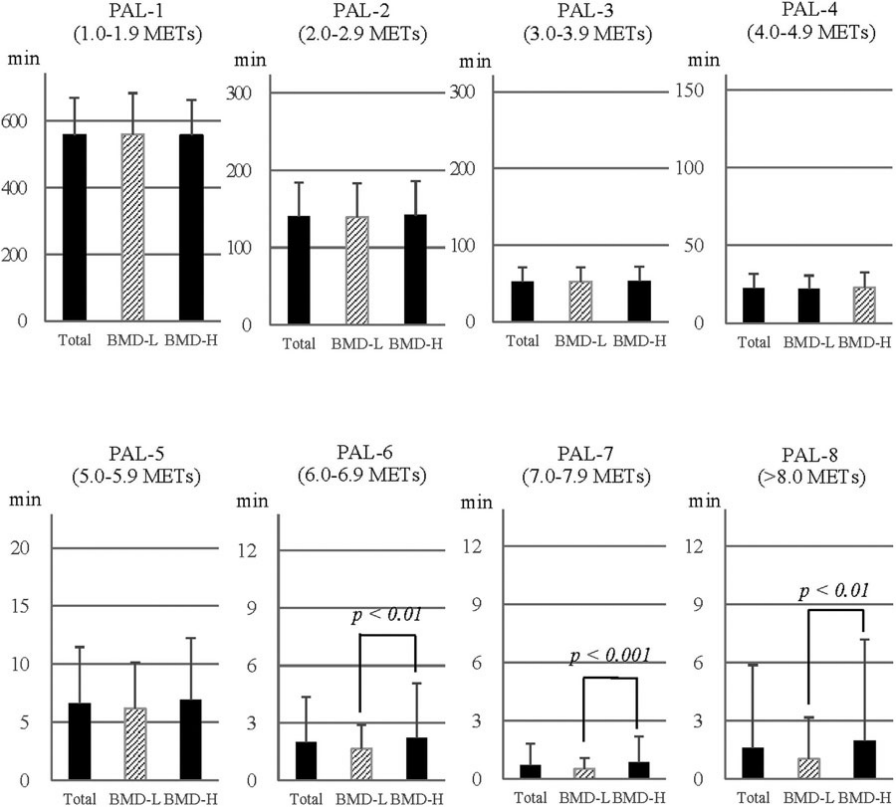
Intensity level of PA. BMD, bone mineral density; PAL, physical activity level.

There were no significant differences in intensity between the two groups in PAL-1 to PAL-5. However, moderate-intensity exercise or longer PA time were significantly more frequent in the BMD-H group than in the BMD-L group, with PAL-6 at 34.8% (*p* < 0.01), PAL-7 at 58.3% (*p* < 0.001), and PAL-8 at 93.2% (*p* < 0.01) (Fig. 1).

## Discussion

This study aimed to determine the effects of body composition and PAL on BMD in healthy young women and to create a baseline values for body composition and PAL required for healthy bone mass. The results suggest that body composition may be related to physical fitness and strength, and daily PA of at least moderate intensity may contribute to higher BMD.

Bones reach PBM around 20 years of age but begin to decrease in mass and density due to factors such as aging, exercise habits, and nutritional status.^30–33^ Bones are readily affected by sex hormones, and age of menarche and menstrual status are believed to have a significant impact on the maintenance or improvement of BMD.^7,8,22^ Furthermore, since bones in women are substantially affected by changes in sex hormones associated with menopause, reaching a high PBM before adolescence is critical.

Previous studies have reported an association between body composition and BMD,^17–22^ and individuals with greater physical abilities often have higher bone density.^5,12,39,40^ Bone density is also positively correlated with muscle or LBM and negatively correlated with adipose body mass.^19–22^ Body composition factors related to physical fitness and strength such as LBM and SMI were significantly higher in the BMD-H group, reflecting the results of previous studies.^5,12,17–22,39,40^

In contrast, although there was no significant difference in the %FAT or total amount of body fat between the two groups, there was a significant difference in BW and BMI. In addition, it is unlikely that BMD is influenced by age-related factors, given that the subjects of this study were healthy young women around the age of 20 with YAM >80%. Thus, muscle mass-related body composition, more so than fat-related body composition, may have more of an effect on healthy BMD in young women around 20 years of age.

Epidemiological studies related to bones or walking have reported that BMD is higher in individuals with faster walking speed.^41,42^ However, age-related factors may have influenced BMD measurements in these studies, which included a wide age range of subjects and focused on gait speed and BMD. Although there were no significant differences between the two groups in this study in terms of walking time or number of steps, the BMD-H group had higher PA (exercise) and calorie expenditure (exercise calorie and total calorie) than the BMD-L group.

These results suggest that the synergistic effect of higher muscle mass and more intense PA in the BMD-H group may have contributed to higher energy expenditure. In other words, a high metabolic rate in young women may be necessary for high BMD.

MS is necessary to improve or maintain healthy trabecular bone, and high-impact exercise loading such as jumping is recommended.^4–6^ A high PBM should be attained during adolescence to prevent the reduction of trabecular bone density or other fractures associated with aging. Therefore, training to increase muscular and general physical strength should be practiced from an early age. Internally and externally generated MS required for bone health include loads produced by strain and pressure.^7,8^

Contraction of muscle tissue caused by PA also stimulates bone tissue. These factors influencing bone tissue can be manifested through daily PA as well as exercise, and increased has been reported to have a positive impact on bone health.^18,43–45^ The duration of low-intensity PA, as determined by PAL, was similar in both groups. However, the BMD-H group maintained moderate-intensity PA of 6 METs or 34.8% higher than that of the BMD-L group.

High-intensity PA and bone mineral quantity are strongly correlated with childhood or pubescent bone characteristics,^18,43^ and a high-intensity PA load is also recommended to improve bone health in middle-aged and older individuals.^44,45^ Although a high-intensity load is effective in improving and maintaining trabecular bone, excessive exercise has been reported to negatively affect bone health.^7,8^ Our results indicate that the bone response to an external load may depend on LBM and amount of PA (age, physical strength, and amount of PA). As such, load intensity adapted to one's individual bone condition may help improve or maintain trabecular bone health.

All subjects in this study were healthy young women with a BMD of >80% of YAM and/or reaching PBM. In contrast, 24.2% of all subjects had exercise habits, and it is difficult to say whether MS, which is required for healthy bones, is dependent only on exercise. In other words, it is reasonable to interpret that the MS required for bones is compensated by all PA in daily life, including exercise.

Thus, the MS load necessary for bones could be obtained from nonexercise PA in daily life, and our results suggest that a more effective load of MS is moderate-intensity PA of >6.0 METs. Given the aforementioned, the baseline values of body composition and PA presented in this study may be useful as baseline values for the acquisition of higher PBM and BMD in healthy young women.

## Conclusion

In conclusion, healthy young women in their early 20s with high BMD tended to have higher muscle mass and BMR, and could endure longer daily PA of moderate intensity or higher than their peers with low BMD. Daily PA >6.0 METs may be beneficial for healthy BMD and PBM in young women.

## Abbreviations Used

%FAT: body fat percentage
BMD-H: bone mineral density-high
BMD-L: bone mineral density-low
BMI: body mass index
BMR: basal metabolic rate
BUA: broadband ultrasound attenuation
BW: body weight
LBM: lean body mass
MET: metabolic equivalent
MS: mechanical stress
OSI: Osteo-Sono Assessment Index
PA: physical activity
PAL: physical activity level
QUS: quantitative ultrasound
SMI: skeletal muscle mass index
SOS: sound of speed
YAM: young adult mean
